# Basal encephalocele in an adult patient presenting with minor anomalies: a case report

**DOI:** 10.1186/1752-1947-8-24

**Published:** 2014-01-27

**Authors:** Naoyuki Harada, Masaaki Nemoto, Chikao Miyazaki, Kosuke Kondo, Hiroyuki Masuda, Jun Nomoto, Nobuo Sugo, Takao Kuroki

**Affiliations:** 1 Department of Neurosurgery (Omori), School of Medicine, Faculty of Medicine, Toho University, 6-11-1, Omori-nishi, 143-8541, Ota-ku, Tokyo, Japan; 2 Department of Neurosurgery, Misato Central General Hospital, Misato, Japan; 3 Department of Neurosurgery (Sakura), School of Medicine, Faculty of Medicine, Toho University, Tokyo, Japan

**Keywords:** Adult, Basal encephalocele, Hypertelorism, Minor anomaly, Strabismus

## Abstract

**Introduction:**

Basal encephalocele is rare in adults. Congenital and acquired cases have been reported with regard to the developmental mechanism, and the pathology has not been elucidated in detail.

**Case presentation:**

We encountered an adult with basal encephalocele strongly suggesting congenital development because of the presence of minor anomalies: strabismus and ocular hypertelorism. The disease manifested as persistent spontaneous cerebrospinal fluid rhinorrhea and repeated meningitis in a 66-year-old Japanese man. On computed tomography, brain tissue protruded through a part of the ethmoid bone of his right anterior skull base, and it was diagnosed as transethmoidal-type basal encephalocele. Regarding his facial form, the distance between his bilateral eyeballs was large compared to his facial width, and his canthal index (defined as inner to outer inter canthal ratio × 100) was calculated as 38.5, based on which it was judged as ocular hypertelorism. In addition, his right eyeball showed strabismus. A right frontotemporal craniotomy was performed for spontaneous cerebrospinal fluid rhinorrhea, and the defective dura mater region was patched with temporal fascia.

**Conclusions:**

Mild minor anomalies that require no treatment are overlooked in adults, but the presence of several anomalies increases the possibility of congenital disease. Therefore, it may be necessary to examine minor anomalies in cases of adult basal encephalocele when considering the possibility that the disease may be congenital.

## Introduction

Basal encephalocele is an uncommon congenital malformation occurring in one in 35,000 to 40,000 live births [1]. Brain tissue protrudes through bony defects in the cribriform plate and body of the sphenoid or ethmoid. Based on the anatomical position of these bony defects, the disease is roughly divided into transethmoidal and transsphenoidal types [2]. Several mechanisms have been proposed to explain the occurrence of basal encephalocele. The most widely accepted theory proposes the occurrence of neuroschisis after neural tube closure [2]. Thus, basal encephalocele is often complicated by midfacial malformation and minor anomalies, such as hypertelorism, anterior cranium bifidum, nasal cleft, cleft lip, cleft palate, and bifid uvula, and most cases are diagnosed at birth [2,3]. By contrast, basal encephalocele diagnosed in adulthood is difficult to diagnose in childhood because abnormalities of the countenance are lacking, and it is incidentally found with spontaneous cerebrospinal fluid rhinorrhea in many cases [1,4-9]. Acquired cases of basal encephalocele have been reported in adults [10]; the pathology of basal encephalocele in adults has not yet been elucidated.

We encountered an adult with a basal encephalocele strongly suggesting congenital development because of the presence of minor anomalies: strabismus and ocular hypertelorism.

## Case presentation

**Figure 1:**
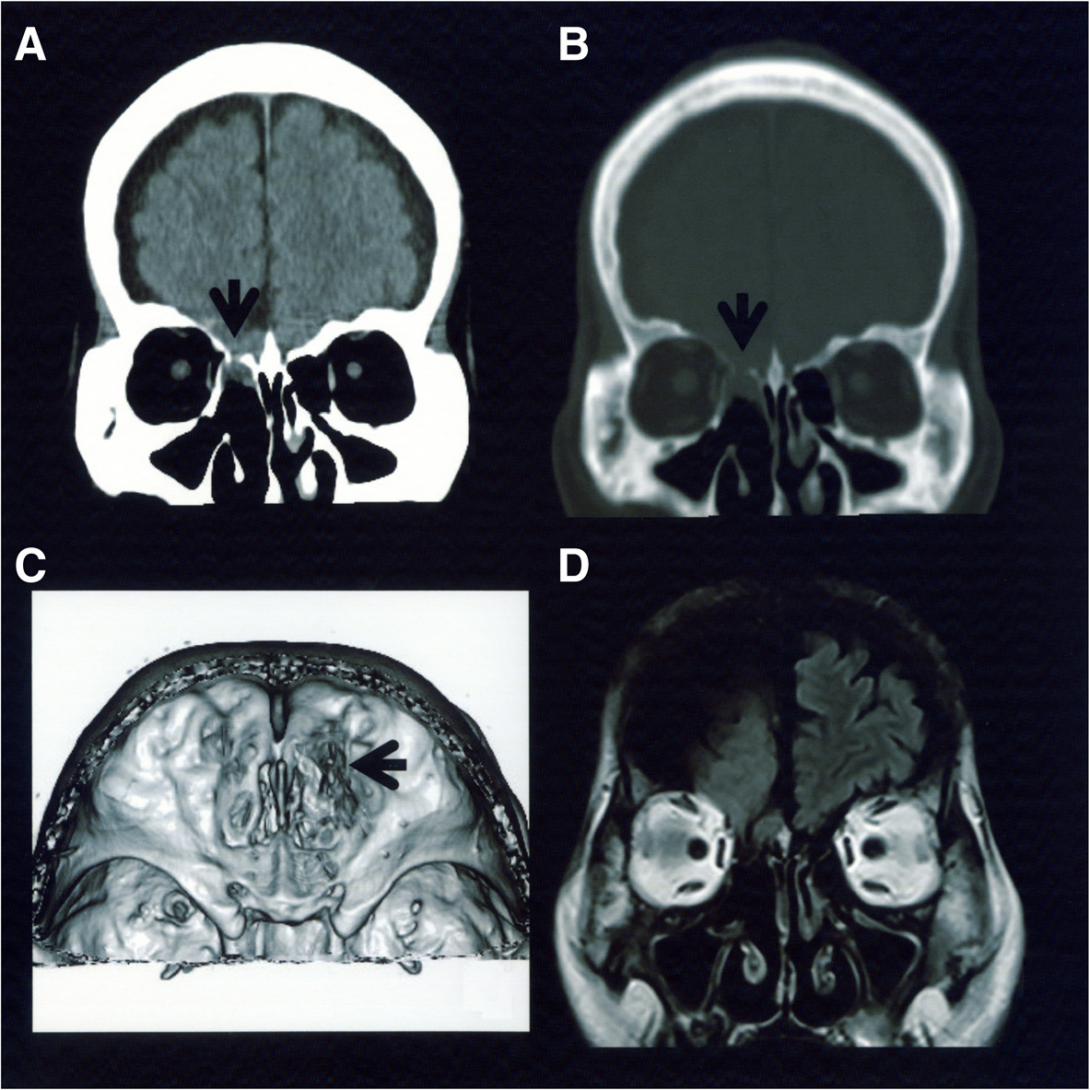
**Preoperative neuroradiological examinations. A**: Coronal section of computed tomography. An isodense mass protruded through a part of the right ethmoid bone (arrow). **B**: Bone window computed tomography. A bone defect was noted in the corresponding region (arrow). **C**: Three-dimensional computed tomography. A round bone defect was present in the ethmoid bone (arrow). **D**: Magnetic resonance imaging. An isodense mass protruding through the ethmoid bone and a partial cystic component were noted.

**Figure 2:**
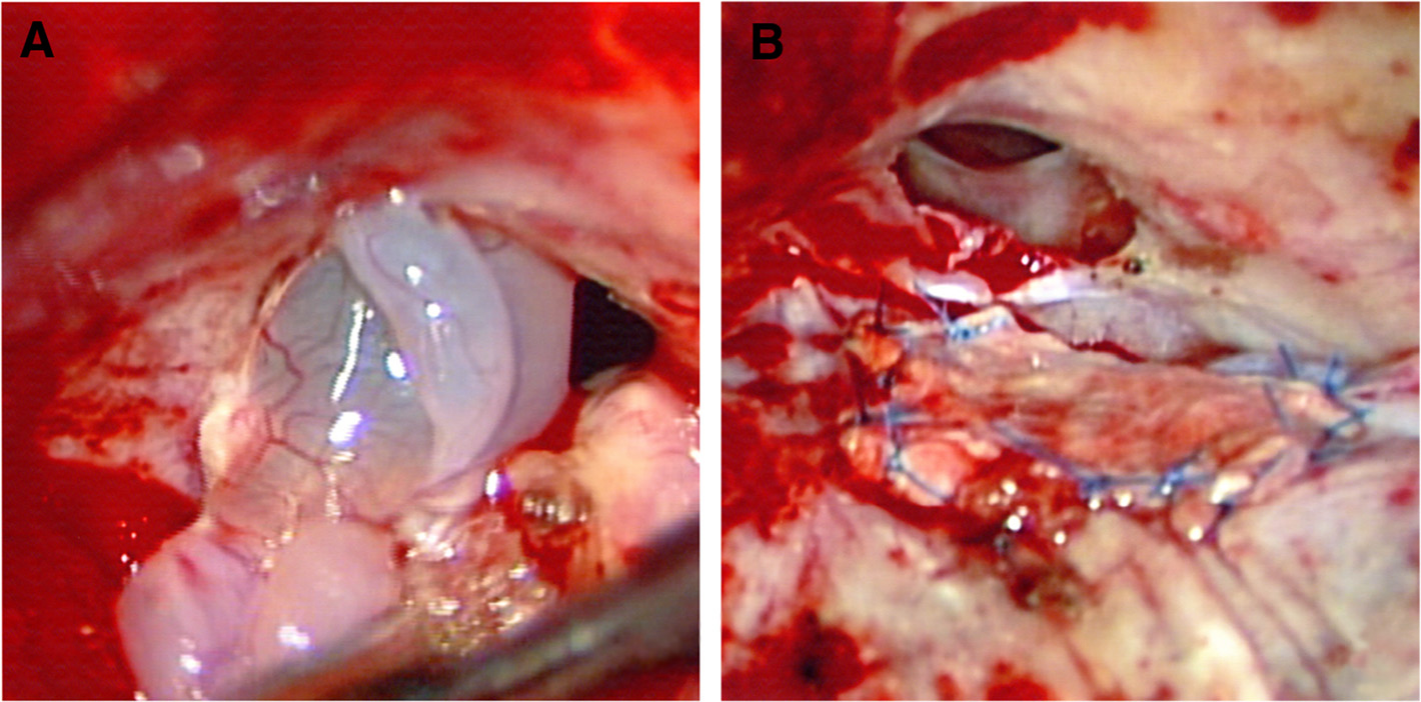
**Surgical photograph. A**: Brain tissue and a cystic component protruded into the paranasal sinus through the right ethmoid bone defect. **B**: After resection of the protruding brain tissue, the defective dura mater region was patched with an unvascularized temporal fascia flap.

The patient was a 66-year-old Japanese man of normal intelligence who had a normal social life and for whom an anomaly had not previously been observed. His height was 161cm, body weight was 50kg, and body mass index was 19.3. He visited a hospital for chief complaints of persistent nasal discharge and fever, and was diagnosed with meningitis and admitted. Because his meningitis repeated, he was transferred to our hospital. On admission, he was conscious and alert, and no neurological deficit was noted other than spontaneous cerebrospinal fluid rhinorrhea and a stiff neck. Regarding his facial form, the distance between his bilateral eyeballs appeared large compared to his facial width. When they were actually measured, his inner and outer canthal distances were 35 and 9mm, respectively, and his canthal index (defined by the inner to outer inter-canthal ratio × 100) [11] was 38.5, based on which his condition was judged as ocular hypertelorism. In addition, strabismus of his right eyeball was noted, and this had been present since infancy. On funduscopy, evaluation was impossible due to a bilateral cataract. No other anomaly was noted. Although a cerebrospinal fluid culture test was negative on admission, the cell count was 1167/μL (monocytes: 292, polycytes: 875); protein level, 413mg/dL (normal value: 10 to 40); and glucose level, 45mg/dL (normal value: 50 to 75), showing the features of bacterial meningitis. An isodense mass protruding through a part of the ethmoid bone of his right anterior skull base was noted in the coronal section on computed tomography (CT) (Figure 1A), and a bone defect was present in the corresponding region on bone window CT (Figure 1B). A round bone defect with a 10mm diameter was noted in the ethmoid bone of his right anterior skull base on three-dimensional CT (Figure 1C), and an isodense mass protruding through the ethmoid bone and a partial cystic component were also observed on magnetic resonance imaging (Figure 1D). Based on the presence of a bony defect in the lateral lamina of the ethmoid bone, the patient was diagnosed with transethmoidal-type basal encephalocele. Spinal drainage and antibiotic administration were performed as initial treatment, and surgery was performed after the meningitis had improved. Right frontotemporal craniotomy was applied, and the epidural space of his anterior skull base was removed. Brain tissue and its cystic component protruded into his paranasal sinus through the defective region of the ethmoid bone (Figure 2A). Dura mater was absent in the defective bone region. After resecting the protruding brain tissue, the defective dura mater region was patched with an unvascularized temporal fascia flap (Figure 2B). The ethmoid bone defect was closed with a small bone fragment prepared by partially dividing the inner table in the frontotemporal craniotomy. The postoperative course was uneventful, and his spontaneous cerebrospinal fluid rhinorrhea was resolved. On pathological examination, the resected specimen was confirmed to be normal brain.

## Discussion

The cause of spontaneous cerebrospinal fluid rhinorrhea in adults may be congenital or acquired [10,12]. Other than trauma, skull base dehiscence that occurs with aging and pneumatization of the paranasal sinus has been reported to be the developmental mechanism of acquired cases [10]. Overweightness or obesity has also been reported to be a factor elevating intracranial pressure and increasing the risk of spontaneous cerebrospinal fluid rhinorrhea [10]. Spontaneous cerebrospinal fluid rhinorrhea-associated secondary meningitis is an important symptom of adult basal encephalocele. A literature review revealed that seven patients with a basal encephalocele, including our patient, also had some congenital anomalies, showing that the frequency was not necessarily low (Table 1). When these congenital anomalies were classified into ophthalmologic findings, midfacial anomalies, and other abnormalities, abnormalities associated with ophthalmologic findings were the most frequently observed. Optic disc abnormalities were noted in four of five patients with ophthalmologic findings, and one was diagnosed with morning glory syndrome [8]. In this syndrome, the optic nerve head is unilaterally funnel-shaped, contains a central white point of glial tissue, and is surrounded by an elevated annulus of chorioretinal pigment disturbance. Since this syndrome originates from failed closure of the embryonic choroid fissure, a large number of ocular and craniofacial abnormalities, such as basal encephalocele, hypertelorism, and cleft palate and lip, have been associated with this syndrome in children [8]. Strabismus on the ipsilateral side of basal encephalocele was noted in two patients including our patient. Strabismus is a minor anomaly and accompanies congenital malformation of the optic disc in many cases [11]. It has been pointed out that basal encephalocele and morning glory syndrome should be suspected when the ophthalmic sign of strabismus is present in infants [8]. However, there is no occasion for actively performing funduscopy in infancy for unilateral and mild cases, and early diagnosis is not necessarily easy. Strabismus had been present in this patient since infancy, but no detailed examination had been performed. In general, strabismus tends to be overlooked until adulthood.

**Table 1:** Adult patients presenting with some anomalies

	**Author**	**Age**	**Sex**	**Side**	**Type of basal encephalocele**	**Spontaneous CSF rhinorrhea**	**Meningitis**	**Ophthalmologic abnormalities**	**Midfacial anomalies**	**Other abnormalities**	**Operation**
**1**	**Bullard *et al*. (1981)**[4]	**24**	**M**	**Lt**	**Transethmoidal**	**+**	**+**	**Lt-dysplastic coloboma (optic disc dysplasia)**	**-**	**-**	**TC**
**2**	**Yamashita *et al*. (1985)**[9]	**53**	**M**	**Rt**	**Transethmoidal**	**+**	**+**	**Rt-optic disc atrophy**	**-**	**-**	**TC**
**3**	**Kitahara *et al*. (1988)**[6]	**33**	**M**	**Lt**	**Transethmoidal**	**+**	**-**	**-**	**-**	**Cerebral palsy**	**TC**
**4**	**Kubo *et al*. (2005)**[1]	**69**	**F**	**Lt**	**Transethmoidal**	**-**	**-**	**-**	**-**	**Multiple angioma in lip and orbit**	**TC**
**5**	**Hasegawa *et al*. (2007)**[5]	**22**	**F**	**Rt**	**Transsphenoidal**	**+**	**+**	**Rt-optic disc atrophy**	**-**	**Rt-ICA occlusion, scleroderma in the right forehead**	**TC, encephalo-arterio-synangiosis at age 6 years**
**6**	**Sasani *et al*. (2009)**[8]	**26**	**M**	**Rt**	**Transsphenoidal**	**+**	**-**	**R-optic disk anomaly (morning glory syndrome), rt-strabismus**	**-**	**-**	**Endoscopic endonasal**
**7**	**Present case**	**66**	**M**	**Rt**	**Transethmoidal**	**+**	**+**	**Rt-strabismus**	**Hypertelorism**	**-**	**TC**

*Abbreviations*: CSF; cerebrospinal fluid; F, female; ICA, internal carotid artery; Lt, left; M, male; Rt, right; TC, transcranial approach was performed for repair of the liquorrhea.

No ocular hypertelorism, which is a midfacial anomaly, in adult cases of basal encephalocele has been reported, but this is the most frequently associated malformation in children [2,3]. In a report on the analysis of basal encephalocele in 10 children, ocular hypertelorism was present in all cases [3]. In addition, the incidence of ocular hypertelorism was high in 22 patients with anterior cranial fossa encephalocele within 11 months after birth (73%) [2]. Three mechanisms have been suggested for the development of ocular hypertelorism [13]. The first mechanism is early ossification of the lesser wings of the sphenoid, and the second one is failure in nasal capsule development allowing the primitive brain vesicle to protrude into the space normally occupied by the capsule, resulting in morphokinetic arrest in the position of the eyes. The third mechanism is a disturbance in the development of the skull base as in craniosynostosis syndromes (such as in Apert or Crouzon syndromes) or midfacial malformation. Ocular hypertelorism is defined as an increase between the inner and outer canthal distances [11]. The canthal index is defined as the inner to outer inter canthal ratio × 100 [11]. The index is higher than 42 in hypertelorism in North American Caucasians [11]. However, it is important to consider physiological ethnic variations in evaluation of the orbital features [14]. In Japan, the canthal index value to diagnose hypertelorism is specified as 38 or higher [13]. Dysmorphology including ocular hypertelorism is a basis to diagnose congenital malformation, but the criteria for dysmorphology are unclear, and many cases are difficult to diagnose based on the value. In particular, the standards of somatometric values are lacking in Japanese, aggravating the situation. Clinical assessment of craniofacial features is based on subjective clinical evaluation of the whole face, but objective measurement is important to confirm the clinical impression [11]. In our patient, a large distance between his bilateral eyeballs was suspected on the first consultation, and his canthal index was actually 38.5, based on which it was judged as ocular hypertelorism. Minor anomalies are mild, requiring no treatment, and are likely to be overlooked in adults. However, the presence of several minor anomalies increases the possibility of congenital disease. An ophthalmologic finding, strabismus, and a midfacial anomaly, ocular hypertelorism, were present in this patient, strongly suggesting congenital basal encephalocele.

## Conclusion

Minor anomalies should be examined in adult basal encephalocele when considering whether the disease may be congenital.

## Consent

Written informed consent was obtained from the patient for publication of this case report and accompanying images. A copy of the written consent is available for review by the Editor-in-Chief of this journal.

## Competing interests

The authors declare that they have no competing interests.

## Authors’ contributions

NH and NS prepared the majority of the manuscript. MN and KK also contributed significantly to the manuscript and participated in the surgical procedures. CM and TK reviewed the manuscript. HM and JN were the attending surgeons. All authors read and approved the final manuscript.
